# Assessing the relationship between gravidity and placental malaria among pregnant women in a high transmission area in Ghana

**DOI:** 10.1186/s12936-022-04252-0

**Published:** 2022-08-20

**Authors:** Ayodele Akinnawo, Kaali Seyram, Ellen Boamah Kaali, Samuel Harrison, David Dosoo, Matthew Cairns, Kwaku Poku Asante

**Affiliations:** 1 London School of Hygiene and Tropical Medicine, London, UK; 2 Research and Development Division, Kintampo Health Research Centre, Ghana Health Service, Kintampo North Municipality, Ghana

**Keywords:** Placental malaria, Gravidity, Primigravidae, Secundigravidae, Multigravidae, Transmission, Regression model

## Abstract

**Background:**

Malaria infection during pregnancy can cause significant morbidity and mortality to a pregnant woman, her fetus and newborn. In areas of high endemic transmission, gravidity is an important risk factor for infection, but there is a complex relationship with other exposure-related factors, and use of protective measures. This study investigated the association between gravidity and placental malaria (PM), among pregnant women aged 14–49 in Kintampo, a high transmission area of Ghana.

**Methods:**

Between 2008 and 2011, as part of a study investigating the association between PM and malaria in infancy, pregnant women attending antenatal care (ANC) clinics in the study area were enrolled and followed up until delivery. The outcome of PM was assessed at delivery by placental histopathology. Multivariable logistic regression analyses were used to investigate the association between gravidity and PM, identify other key risk factors, and control for potential confounders. Pre-specified effect modifiers including area of residence, socio-economic score (SES), ITN use and IPTp-SP use were explored.

**Results:**

The prevalence of PM was 65.9% in primigravidae, and 26.5% in multigravidae. After adjusting for age, SES and relationship status, primigravidae were shown to have over three times the odds of PM compared to multigravidae, defined as women with 2 or more previous pregnancies [adjusted OR = 3.36 (95% CI 2.39–4.71), N = 1808, P < 0.001]. The association appeared stronger in rural areas [OR for PG vs. MG was 3.79 (95% CI 3.61–5.51) in rural areas; 2.09 (95% CI 1.17–3.71) in urban areas; P for interaction = 0.07], and among women with lower socio-economic scores [OR for PG vs. MG was 4.73 (95% CI 3.08–7.25) amongst women with lower SES; OR = 2.14 (95% CI 1.38–3.35) among women with higher SES; P for interaction = 0.008]. There was also evidence of lower risk among primigravidae with better use of the current preventive measures IPTp and LLIN.

**Conclusions:**

The burden of PM is most heavily focused on primigravidae of low SES living in rural areas of high transmission. Programmes should prioritize primigravidae and young women of child-bearing age for interventions such as LLIN distribution, educational initiatives and treatment to reduce the burden of malaria in first pregnancy.

**Supplementary Information:**

The online version contains supplementary material available at 10.1186/s12936-022-04252-0.

## Background

Malaria infection during pregnancy can cause significant morbidity and mortality to a pregnant woman, her fetus and newborn [1]. A common complication is placental malaria (PM), in which *Plasmodium falciparum* infects erythrocytes and cause them to sequester within the placental intervillous space [2]. Mothers infected with PM are at risk of adverse perinatal outcomes including miscarriages, pulmonary oedema, hypoglycaemia and maternal anaemia [3]. Adverse birth outcomes for the fetus include spontaneous abortion, still birth, intra uterine growth retardation, preterm delivery and low birth weight [4, 5].

Globally, malaria infection affects approximately 11 million pregnancies-predominantly in the sub-Saharan African region, and up to 100,000 infant deaths are attributed to PM every year [6]. In regions of low-transmission, levels of acquired immunity to malaria tend to be low, and all pregnant women are vulnerable to malaria, which is often symptomatic [1]. In contrast, in regions of endemic transmission, levels of acquired immunity tend to be high and *P. falciparum* infection tends to be asymptomatic during pregnancy [1, 7]. The World Health Organization (WHO) recommends women in high-transmission areas be given long-lasting insecticidal nets (LLINs), at least three doses of intermittent preventative therapy with sulfadoxine-pyrimethamine (IPTp-SP) and prompt diagnosis and treatment [1, 8].

Susceptibility to malaria increases during pregnancy [9]. In high-transmission areas, primigravidae, are at substantially greater risk of PM than secundigravidae or multigravidae, due to a parity-specific immune mechanism acquired through successive pregnancies [9, 10]. Current understanding is that placental *P. falciparum* parasites express surface antigens on the surface of infected erythrocytes, which facilitate sequestration in the placenta via adhesion to placental chondroitin sulphate A. Primigravidae lack antibodies to block this adhesion, however, over repeated exposure and successive pregnancies, the antibodies increase, making secundigravidae and multigravidae less susceptible to infection [11]. Primigravid women are also at elevated risk from infections acquired prior to conception, because use of protective measures is much lower among adolescents and women of child-bearing age prior to first pregnancy than among women who have been pregnant and received LLIN through ANC [12, 13].

Though the extra risk of PM in primigravidae, compared to other gravidities has been well characterized in the literature, further evidence showing how this risk reduces over successive pregnancies, within a specific setting at a specific time, as well as evidence quantifying the effect of malaria-specific interventions on this risk is necessary. Moreover, some effect modifiers are still controversial, with very few studies investigating the effect of factors such as area of residence, doses of IPTp-SP, ITN use and socio-economic grouping on the relationship between gravidity and PM. This study aims to add to the available literature by quantifying the strength of these associations, and characterizing the changing profile of risk with gravidity in a high-transmission area of Ghana.

Although the elevated risk in primigravidae is well known, how this elevated risk may vary within a particular context, and how risk may be influenced by other factors is less well described. It is also challenging to disentangle the risk from low gravidity itself (i.e., naivety to pregnancy-specific antigenic variants of *P. falciparum*) and from factors associated with gravidity (incl. maternal age, use of protective measures), which might also affect malaria risk. To investigate these issues, the association between gravidity and placental malaria was investigated using data from a large cohort study in Ghana, in which placental malaria status was determined using the gold standard of placental histology, and in which a wide range of factors related to exposure were carefully documented.

## Methods

This secondary data analysis uses data collected during a prospective cohort study, which investigated PM as a risk factor for malaria infection in infants. The study was conducted in the Bono East Region of Ghana [14]. Between 2008 and 2011, forty-two communities were selected from the Kintampo Health and Demographic Surveillance System (KHDSS). All pregnant women residing in the regions were identified via vital registers collated by community key informants or Kintampo Health and Demographic Surveillance System staff who made twice yearly home visits, recording demographic events such as pregnancy, births, deaths, and migration [15, 16]. The only maternal exclusion criteria applied to the above study was that women were required to stay in the study region for one year post delivery. Of the 2160 pregnant women contacted, 337 (15.6%) were excluded due to refused consent, unsuccessful placental tissue collection or stillbirth, resulting in a final cohort of 1823 pregnant women. The final cohort for this secondary analysis included all 1823 women.

Surveys were administered by interviewers at enrolment (i.e., while the woman was still pregnant) and information on demographic, socioeconomic and obstetric characteristics and the PM infection status of women were collected [15]. Upon delivery, a placental biopsy was taken and assessed by histopathologists for the outcome of placental malaria, as described below [15]. Ethical approval for the original study was obtained from the ethics committees of Kintampo Health Research Centre, Ghana Health Service, LSHTM, and Noguchi Memorial Institute for Medical Research. Written informed consent was obtained from all study participants.

### Classification of outcome and primary exposure

Mothers responded to a questionnaire on enrolment, which captured their obstetric history, including the number of all living, deceased, aborted or ectopic babies born to the mother. The total number was calculated (minus the current pregnancy) to give the gravidity number, confirmed after cross-checking with the woman. To ascertain the PM status, women were followed-up until delivery, at which time a placental biopsy was taken. A histopathologist then categorized each sample as no infection, acute infection (presence of parasites only), chronic infection (presence of both parasites and haemozoin pigment), or past infection (presence of haemozoin pigment only), using the classification system described by Bulmer et al. [17]. Acute, chronic and past infections were considered as ‘placental malaria’. A second histopathologist checked 28% of randomly selected samples and the pairwise correlation coefficient between the two readings was 0.92, indicating a high degree of correlation.

### Statistical analysis

Cleaned data were analysed using STATA 16 (StataCorp, College Station, Texas). Socio-economic quintiles (SES) were derived for the study population using principal component analysis, which aggregated information on women’s household amenities, household construction and durable assets (Additional file 1). The crude association between gravidity and placental malaria was determined, and the effect of adjusting for potential confounders (i.e., factors related to both gravidity and malaria risk) on this association was explored, firstly using Mantel–Haenszel stratification and then multivariable logistic regression, to derive adjusted odds ratios. Potential effect modifiers pre-specified for investigation a priori were, area of residence (rural vs. urban), SES, IPTp use and ITN use. Evidence for effect modification was assessed using the Likelihood Ratio Test (LRT).

Multicollinearity was assessed by checking the standard error of the coefficient when each confounder was added into the model. One of any two variables found to be highly correlated with each other was either removed from the model or kept in if decided a priori to be an important confounder (or if keeping the variable in the model produced the smallest root mean square error).

## Results

All 1823 participants were aged between 14 and 49, with a mean age of 26.6 years. At delivery, 37.4% (683/1823) of women had the outcome of placental malaria (PM). Of these, 9.4% (64/683) had acute infection, 10.4% (71/683) had chronic infection and 80.2% (548/683) had past infection.

**Fig. 1:**
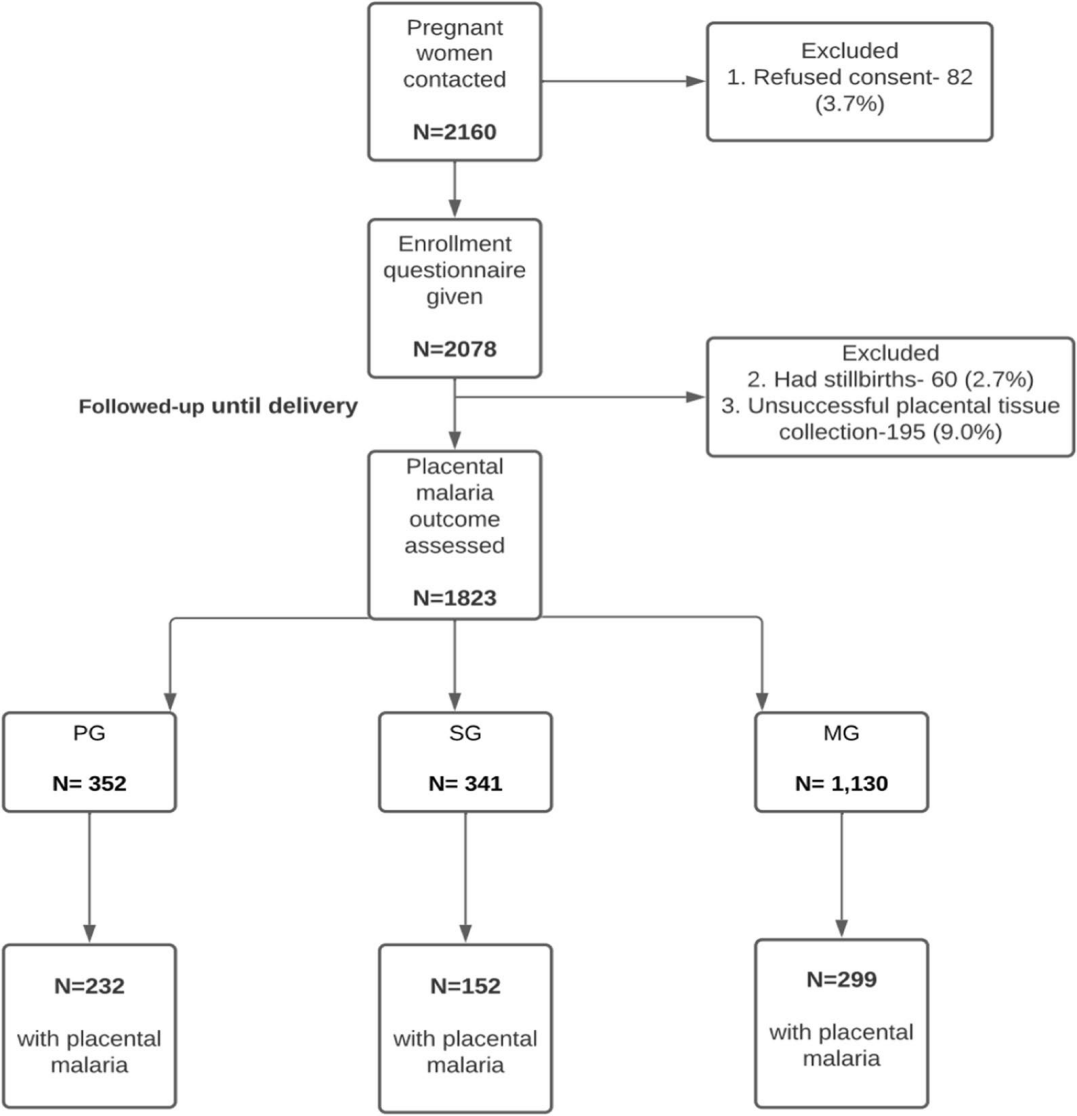
Flowchart to summarize final study participants. *MG* multigravidae; *SG* secundigravidae; *PG* primigravidae

The number of previous pregnancies women had in the study ranged from zero to four. Three hundred and fifty two (19.3%) women were primigravidae and 341 (18.7%) were secundigravidae. Of the 1130 multigravidae, 325 (17.8%) women had experienced two prior pregnancies, 251 (13.8%) women had experienced three prior pregnancies and 554 (30.4%) women had experienced 4 prior pregnancies (Fig. 1; Additional file 2). The risk of developing PM in the full sample of women was 37.5% (683/1823), however the risk was higher in PG, 65.9% (232/352), than SG, 44.6% (152/241), or MG, 26.5% (299/1130).

**Fig. 2:**
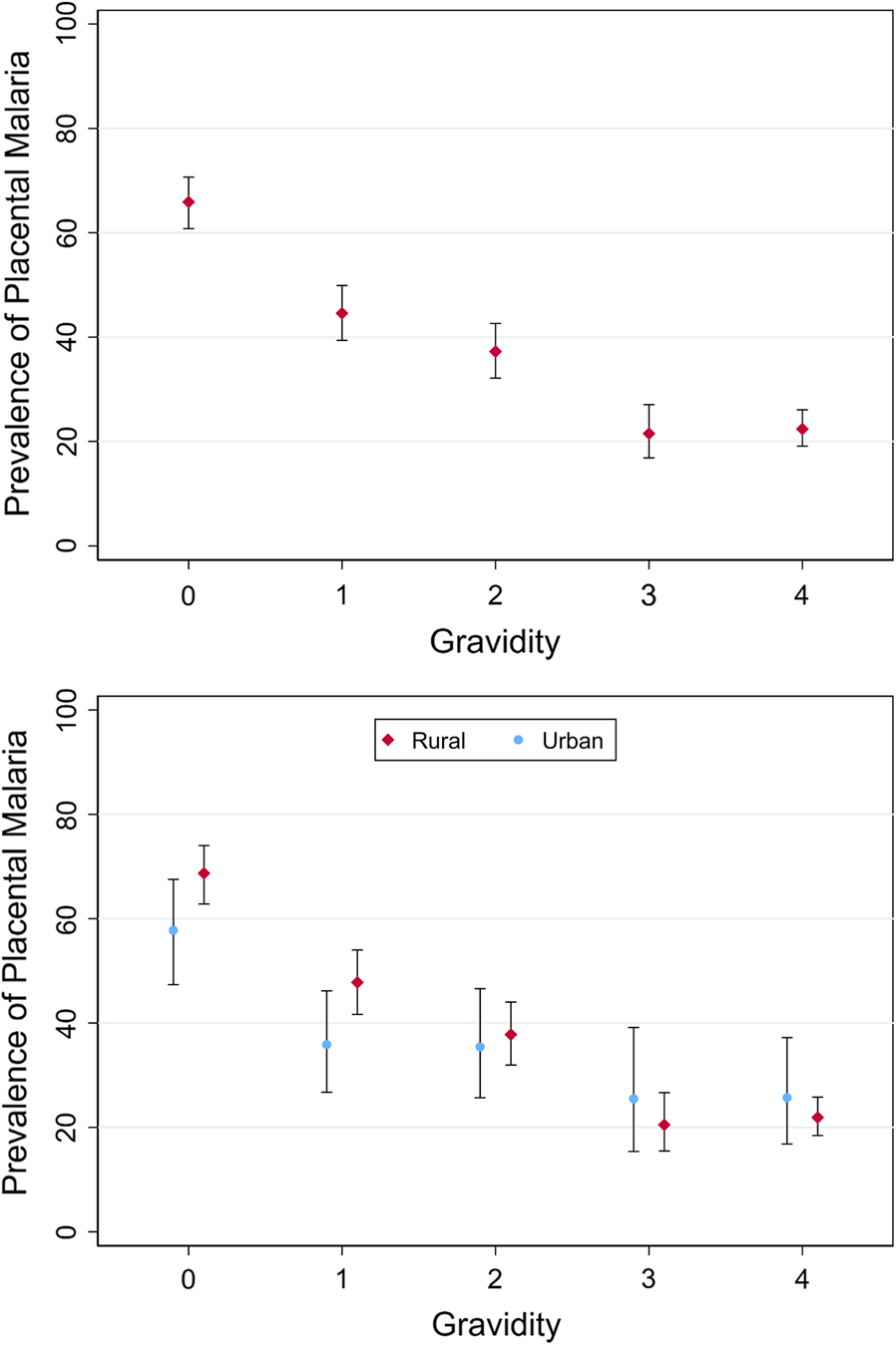
Graphs to show the prevalence of placental malaria infection, and associated confidence intervals in each gravidity group

As gravidity increased, the prevalence of PM decreased sharply over the first three pregnancies (i.e., gravidity 0–2), but was similar between women with 3 or 4 previous pregnancies (Fig. 2a). The univariate analysis (Additional file 3) showed that gravidity was strongly associated with odds of PM (global p-value from LRT < 0.001), with evidence that the association was non-linear (P-value for departure from linearity P = 0.0017). The decrease in prevalence with increasing gravidity was less marked among women living in urban areas than among women living in rural areas (Fig. 2b).

For multivariable analysis, to avoid data sparsity, gravidity was grouped into three levels: primigravidae (PG), secundigravidae (SG) and multigravidae (2 or more pregnancies, MG). Comparing PG to MG, the crude odds ratio for PM was OR = 5.37 95%, CI 4.08–7.07, P < 0.001, and for SG was OR = 2.24 95%, CI 1.73–2.88, P < 0.001 (Table 1). The final multivariable logistic regression model (Table 1) included PM, gravidity, and the confounding variables age, wealth index and relationship status. After adjusting for all confounding variables, the magnitude of the association diminished, however, there remained strong evidence that primigravidae were at increased odds of developing PM, adjusted OR = 3.36 (95% CI 2.39–4.71), N = 1808, P < 0.001.

**Table 1:** Crude and adjusted odds ratios for the effect of gravidity as a multi-level variable, age, wealth index and relationship status on placental malaria, adjusted for confounders, estimated by logistic regression in Ghanaian women aged 14–49 (N = 1808)

Variable	Category	Crude model		Fully adjusted model^a^	
		OR (95% CI)	P-value (LRT)	OR (95% CI)	P-value (LRT)
Gravidity	MG	(Ref.)	< 0.001	(Ref.)	< 0.001
	SG	2.24 (1.73–2.88)		1.67 (1.24–2.25)	
	PG	5.37 (4.08–7.07)		3.36 (2.39–4.71)	
Age	< 18	(Ref.)		(Ref.)	
	18–25	0.36 (0.26–0.51)		0.66 (0.45–0.96)	
	25–49	0.14 (0.10–0.21)		0.43 (0.27–0.67)	
Wealth index	Least poor	(Ref.)		(Ref.)	
	Less poor	1.12 (0.82–1.53)		1.10 (0.78–1.54)	
	Poor	1.40 (1.02–1.90)		1.48 (1.06–2.07)	
	More poor	2.08 (1.53–2.84)		2.14 (1.53–2.98)	
	Extremely poor	1.59 (1.17–2.17)		1.93 (1.38–2.69)	
Relationship status	Married	(Ref.)		(Ref.)	
	Living together	1.97 (1.60–2.42)		1.25 (0.99–1.59)	
	Widowed/divorced/separated	1.90 (0.98–3.71)		0.91 (0.44–1.87)	
	Single	3.40 (2.36–4.88)		1.38 (0.91–2.09)	

*LRT* Likelihood ratio test, *MG* multigravidae; *PG* primigravidae

^a^Model adjusted for age group, wealth index and relationship status

Effect modification (interaction) was explored in the adjusted model (Table 2). The association between primigravidity and PM was stronger in rural areas (adjusted OR = 3.79, 95% CI 3.61–5.51), compared to urban areas (adjusted OR = 2.09, 95% CI 1.17–3.71), LRT p-value P = 0.07. The five category wealth index was collapsed into two categories (grouping women in the upper 2 quintiles and lower 3 quintiles) when assessing effect modification, in order to increase the power of the test for interaction. The OR comparing PG to MG was 4.73 (95% CI 3.08–7.25) among women in the lower three quintiles, and 2.14 (95% CI 1.38–3.35) among women in the upper two quintiles, LRT P = 0.008.

**Table 2:** Stratum-specific odds ratios of effect modifiers to the association of gravidity as a multi-level variable and placental malaria, among Ghanaian mothers aged 14–49, estimated by logistic regression fitted with interaction parameters after adjustment^a,b^ for confounders

Variable	Category	Gravidity group	% PM (pm/N)	Stratum specific OR (95% CI)	P-value (LRT)
Stratified by area (N = 1823)	Urban	Multigravidae	29.5% (59/200)	1^a^	0.07
		Secundigravidae	35.9% (33/92)	1.04 (0.60–1.81)^a^	
		Primigravidae	57.7% (52/90)	2.09 (1.17–3.71)^a^	
	Rural	Multigravidae	25.8% (240/930)	1^a^	
		Secundigravidae	47.8% (119/249)	1.87 (1.34–2.62)^a^	
		Primigravidae	68.7% (180/262)	3.79 (3.61–5.51)^a^	
Stratified by wealth index (N = 1823)	Higher	Multigravidae	23.1% (98/424)	1^b^	0.008
		Secundigravidae	32.9% (51/155)	1.21 (0.79–1.86)^b^	
		Primigravidae	52.0% (78/150)	2.14 (1.38–3.35)^b^	
	Lower	Multigravidae	28.5% (201/706)	1^b^	
		Secundigravidae	54.3% (101/186)	2.05 (1.41–2.98)^b^	
		Primigravidae	76.2% (154/202)	4.73 (3.08–7.25)^b^	
Stratified by ITN use (N = 1782)	Don’t use	Multigravidae	23.9% (137/573)	1^a^	0.10
		Secundigravidae	43.3% (62/143)	1.83 (1.20–2.79)^a^	
		Primigravidae	70.0% (100/145)	4.22 (2.68–6.67)^a^	
	Use	Multigravidae	29.6% (158/532)	1^a^	
		Secundigravidae	45.6% (87/191)	1.39 (0.95–2.04)^a^	
		Primigravidae	62.6% (124/198)	2.36 (1.54–3.60)^a^	
Doses of Fansidar (IPTp) (N = 1820)	0	Multigravidae	16.1% (10/62)	1	0.12
		Secundigravidae	38.1% (8/21)	2.00 (0.63–6.27)	
		Primigravidae	69.2% (9/13)	7.13 (1.76–28.81)	
	1	Multigravidae	26.6% (37/139)	1	
		Secundigravidae	38.6% (17/44)	1.28 (0.61–2.69)	
		Primigravidae	78.4% (40/51)	5.50 (2.46–12.32)	
	2	Multigravidae	25.5% (72/282)	1	
		Secundigravidae	48.3% (42/87)	2.09 (1.22–3.56)	
		Primigravidae	71.0% (64/90)	4.86 (2.71–8.73)	
	3	Multigravidae	27.8% (179/645)	1	
		Secundigravidae	45.0% (85/189)	1.56 (1.07–2.28)	
		Primigravidae	60.4% (119/197)	2.38 (1.59–3.57)	

*LRT* Likelihood ratio test

^a^Adjusted for age, wealth and relationship status

^b^Adjusted for age relationship

Comparing PG to MG within strata of bed net use, the association with placental malaria was stronger among non-users of ITNs, OR = 4.22, (95% CI 2.68–6.67, P = 0.10) than among primigravidae who did use ITNs (adjusted OR = 2.36, 95% CI 1.54–3.60), LRT P = 0.01. Finally, there was some suggestion that the increased odds of PM in PG, relative to MG, was greatest in primigravidae who took no IPTp-SP (OR = 7.13, 95% CI 1.76–28.81), and smallest in primigravidae who took at least 3 IPTp-SP (OR = 1.86, 95% CI 2.38 (1.59–3.57), although the confidence intervals around the ORs in the strata of IPTp uptake overlapped, LRT p-value, P = 0.12).

A slight degree of multicollinearity was found between age and gravidity as a binary variable. As the model with all confounding variables had the smallest root mean square error, age was left in the model despite the slight degree of collinearity with gravidity and this model was used as the final model. No other confounders were shown to be multicollinear with each other when put into the final model.

## Discussion

### Summary of results

This study aimed to explore the relationship between gravidity and PM among women in a high transmission area of Ghana, and explore how this risk was modified by other characteristics of the pregnant women. After adjustment for age, wealth index and relationship status through a multivariable logistic regression model, there was strong evidence that, primigravidae were at markedly higher risk of developing PM than multigravidae. Importantly, evidence was found that the extent to which primigravidae were at an elevated risk was influenced by area of residence and relative wealth, with approximately 75% of primigravid women in rural areas experiencing PM, compared to 30% of multigravidae living in urban areas. Use of protective measures such as ITNs and IPTp-SP may mitigate this risk.

The strong overall association between gravidity and PM was expected given the biological mechanism behind acquired immunity. From the analyses of gravidity as a multi-level variable, it can be seen that the excess risk in PG declines rapidly with successive pregnancies. In a higher-transmission area like Kintampo North Municipality, this is likely to occur more quickly than in a lower transmission area, because immunity to PM should be acquired over a smaller number of pregnancies [10, 18, 19]. However, few studies have investigated how the effect of gravidity on PM varies according to level of exposure (assessed here through the proxies of place of residence and SES) and use of protective measures. Comparable results to those presented were obtained treating gravidity as a binary variable (grouping SG with MG), in order to increase power (Additional file 4).

Relative to multigravidae, the increase in risk in primigravidae was greater in rural locations, and in women of lower SES, than in urban locations and women of higher SES. These result are consistent with both rural residence and poverty being strongly associated with elevated malaria risk (e.g., [10, 20]), and could potentially lead to very large differences in individual risk within a small geographic area. For example, 75.6% of primigravidae in the lowest SES stratum, living in rural areas had PM, compared to 30.6% of multigravidae in urban areas, in the highest SES group. Exposure to higher infection rates will lead to faster acquisition of immunity over successive pregnancies and a sharper drop in infection rates with increasing gravidity. A further contributing factor, (although this was not investigated in this study) is that there may be higher prevalence of infection prior to first pregnancy in adolescents and young women of low SES in rural areas. Although the data reflects the situation around 10 years ago, the differences in transmission between rural and urban areas, and the different relationship between gravidity and prevalence in areas with different levels of transmission provide insights into the potential changes that may arise from future reductions in transmission in rural areas. If the finding of effect modification is true, then acquisition of immunity may occur more slowly as transmission is brought under control, and the excess risk in PG compared to MG may become smaller. This is a positive change, but will mean that women of higher gravidities may bear a larger share of the burden of PM, and prioritisation of women of all gravidities may need to become the focus.

The finding that current preventive interventions for malaria in pregnancy (ITN use and uptake of IPTp-SP) appear to mitigate the excess risk in primigravidae is consistent with the demonstrated effectiveness of these interventions [1]. PG using an ITN were still at increased odds of PM compared to MG who used an ITN, but the odds ratio was much smaller than PG without ITN use compared to MG without ITN use [21]. The ORs comparing PG to MG were progressively smaller with increasing doses of IPTp-SP, i.e. a dose–response relationship, although the confidence intervals were wide in each stratum. Assuming this finding is robust, it would support the effectiveness of IPTp-SP in reducing the risk among primigravid women. In Ghana, a minimum of 3 doses, up to a maximum of five doses of IPTp-SP are recommended (one at each ANC visit from the second trimester until delivery), and coverage of three doses is reasonably high at 60% [8, 22]. Although these findings are plausible, evidence for effect modification was less strong than for areas of residence and SES, and there is potential for residual confounding through better access to these interventions likely being a proxy for lower malaria risk.

Strengths of the study include collection of data on a wide range of potential exposures, with a high degree of data completeness. The outcome of PM infection and infection type was objective and diagnosed by histology, which has a high specificity. Key limitations are the potential inaccuracy of self-reported measures such as maternal ITN use, uptake of IPTp-SP (through social-desirability bias) and to a lesser extent education status. There could also be residual confounding either from imperfect measurement of confounders that were included, for example, the use of place of residence and SES as proxies to assess general level of exposure to malaria, or from other factors unaccounted for in the analysis, which could be related to both gravidity and PM. One such possibility is the presence of maternal co-infections, which might be more prevalent in primigravid mothers, and which might influence the progression to PM. In this study, and more generally in studies investigating the effect of gravidity on malaria risk, it would be interesting to know the full exposure history of pregnant women, including in earlier pregnancies (prior to the pregnancy that resulted in recruitment into this study). This would allow disentanglement of previous exposure to malaria in pregnancy (i.e., previous exposure to pregnancy-specific antigenic variants of *P. falciparum*) from increasing gravidity itself (simply having been pregnant previously)*.* However, following women over successive pregnancies in order to measure this would be challenging, as this may require long periods of follow-up. Results should, therefore, be interpreted in light of these limitations. However, residual confounding would need to be strong to negate the effects observed, which is unlikely.

## Conclusion

This study provides evidence that the risk of PM in women in a high transmission area of Ghana is markedly higher among primigravidae than multigravidae. This risk is not shared equally—primigravidae from rural areas, and those with lower relative wealth, who typically have lower access to health services, are likely to be at highest risk. Programmes distributing ITN should ensure that primigravidae in these high risk groups are prioritized for interventions such as ITN distribution, and encouraged to attend ANC as early as possible in their pregnancy to receive IPTp-SP. Given the likelihood that risks among primigravid women reflect risks among the population from which these pregnancies arose, control programmes should also prioritize young women of child bearing age to try to protect first pregnancies from PM.

## Supplementary Information

**Additional file 1: Table S1.** Principal component analysis.

**Additional file 2: Table S2.** Proportion of past, chronic and acute malaria within the different gravidity groups.

**Additional file 3: Table S3.** Baseline distribution of study sample characteristics and crude bivariate associations of placental malaria against different explanatory variables among Ghanaian mothers.

**Additional file 4: Table S4.** Crude and adjusted odds ratios for the effect of gravidity as a binary variable, age, wealth index and relationship status on placental malaria, adjusted for confounders, estimated by logistic regression. **Table S5.** Stratum-specific odds ratios from potential effect modifiers identified through Mantel-Haenszel analysis, after assessing for potential confounders. **Table S6.** Stratum specific odds ratios of effect modifiers of the association between gravidity as a binary variable and placental malaria, estimated by logistic regression fitted with interaction parameters after adjustment for confounders. Gravidity as a binary variable specific results.

## Data Availability

The datasets used and/or analysed during the current study are available from Kwaku Poku Asante on reasonable request.

## Abbreviations

ADJ: Adjusted
CI: Confidence interval
IPTp-SP: Intermittent preventative therapy with sulfadoxine-pyrimethamine
LRT: Likelihood ratio test
M-H: Mantel–Haenszel
MG: Multigravidae
OR: Odds ratio
PG: Primigravidae
PM: Placental malaria
RRR: Relative risk ratio
SES: Socio-economic score
SG: Secundigravidae
