# Supplementary material for: Assessing the relationship between gravidity and placental malaria among pregnant women in a high transmission area in Ghana
Source: Malar J. 2022 Aug 20;21:240. doi: 10.1186/s12936-022-04252-0 (PMC9392271; doi:10.1186/s12936-022-04252-0)
Supplement: Supplementary file 1 — Additional file 1: Table S1. Principal component analysis. [file 12936_2022_4252_MOESM1_ESM.docx]

Additional file 1

Principal Component Analysis

A socio-economic score (SES) was developed by aggregating information on all housing construction, household and durable assets. This score was created using a principal component analysis (PCA) on STATA 16, which created a unidimensional measure derived specifically for the study population. PCA works best when variables are correlated and the distribution varies across individuals and variables- with more unequal distributions across individuals given more weight. Thus, asset variables were chosen by first carrying out a descriptive analysis on all asset variables in the dataset to evaluate means, frequencies and standard deviations and decide which variables to include in the analysis. Due to the high amount of variation, a correlation matrix was used. Categorical variables were coded into binary variables and missing values were explored.

Individuals were then grouped into categories/quintiles, reflecting different SES levels. The range of asset variables included was broad enough to ensure problems relating to clustering – individuals grouped together in a small number of distinct clusters, or truncation- a more even distribution within the SES scale but over narrow range, were avoided.

## Durable and household assets included in the principal components analysis

| **Household characteristics** | | **Mean** | **Standard deviation** | **Component/factor score** |
| --- | --- | --- | --- | --- |
| Water piped into dwelling/yard/plot | | 0.030 | 0.171 | 0.091 |
| Flush latrine / WC | 0.289 | | 0.453 | 0.195 |
| Sole/Joint ownership of house | 0.358 | | 0.480 | -0.074 |
| Cement floor | 0.899 | | 0.302 | 0.151 |
| Metal/asbestos roof | 0.291 | | 0.454 | -0.220 |
| Cement wall | 0.682 | | 0.466 | 0.233 |
| Kitchen in home | 0.528 | | 0.499 | -0.013 |
| Separate sleeping room for children | 0.155 | | 0.362 | 0.087 |
| Domestic in household | 0.035 | | 0.184 | 0.065 |
| Own ITN | 0.545 | | 0.498 | 0.054 |
| ITN use | 0.365 | | 0.482 | -0.002 |
| Salaried professional in household | 0.060 | | 0.238 | 0.111 |
| Land ownership | 0.670 | | 0.470 | 0.015 |
| Farm ownership | 0.879 | | 0.326 | -0.125 |
| Chickens or ducks | 0.584 | | 0.493 | -0.067 |
| Sheep or goat | 0.381 | | 0.486 | -0.002 |
| Cattle | 0.048 | | 0.213 | 0.010 |
| Donkey/Cart | 0.035 | | 0.183 | 0.000 |
| Other animals | 0.267 | | 0.442 | -0.009 |
| Table | 0.895 | | 0.306 | 0.115 |
| Sleeping mattress | 0.879 | | 0.326 | 0.150 |
| Cupboard, wardrobe, room divider | 0.291 | | 0.454 | 0.252 |
| Mosquito net | 0.617 | | 0.486 | -0.002 |
| Insecticide Treated Mosquito Net | 0.561 | | 0.496 | 0.054 |
| radio | 0.804 | | 0.397 | 0.109 |
| TV | 0.312 | | 0.463 | 0.285 |
| Gas or electric cooker | 0.055 | | 0.228 | 0.198 |
| Fridge or freezer | 0.172 | | 0.378 | 0.301 |
| Bicycle | 0.600 | | 0.490 | 0.011 |
| Motorcycle | 0.100 | | 0.300 | 0.116 |
| Tractor | 0.020 | | 0.141 | 0.038 |
| Telephone (fixed/mobile) | 0.741 | | 0.438 | 0.166 |
| Store/shop/kiosk | 0.160 | | 0.367 | 0.187 |
| Commercial Vehicle | 0.054 | | 0.227 | 0.086 |
| Car | 0.023 | | 0.150 | 0.081 |
| Electric iron | 0.189 | | 0.189 | 0.284 |
| Fan | 0.223 | | 0.417 | 0.295 |
| Satellite dish | 0.009 | | 0.093 | 0.066 |
| Computer | 0.040 | | 0.196 | 0.148 |
| Sewing machine | 0.363 | | 0.481 | 0.060 |
| Household electricity | 0.437 | | 0.496 | 0.266 |
