# Supplementary material for: Assessing the relationship between gravidity and placental malaria among pregnant women in a high transmission area in Ghana
Source: Malar J. 2022 Aug 20;21:240. doi: 10.1186/s12936-022-04252-0 (PMC9392271; doi:10.1186/s12936-022-04252-0)
Supplement: Supplementary file 2 — Additional file 2: Table S2. Proportion of past, chronic and acute malaria within the different gravidity groups. [file 12936_2022_4252_MOESM2_ESM.docx]

## Additional file 2

**Proportion of past, chronic and acute malaria within the different gravidity groups.**

|  | **Gravidity** |  |  |  |  |  |  |  |
| --- | --- | --- | --- | --- | --- | --- | --- | --- |
|  | **Primigravidae** |  | **Secundigravidae** | | **Multigravidae** |  | **Total** |  |
| **Infection Status** | No. | % | No. | % | No. | % | No. | % |
| No infection | 120 | 34.1 | 189 | 55.4 | 831 | 73.5 | 1140 | 62.5 |
| Past infection | 175 | 49.7 | 121 | 35.5 | 252 | 22.3 | 548 | 30.1 |
| Chronic infection | 34 | 9.7 | 16 | 4.7 | 21 | 1.9 | 71 | 3.9 |
| Acute Infection | 23 | 6.5 | 15 | 4.4 | 26 | 2.3 | 64 | 3.5 |
| **Total** | **352** |  | **341** |  | **1130** |  | **1823** |  |
