# Supplementary material for: Assessing the relationship between gravidity and placental malaria among pregnant women in a high transmission area in Ghana
Source: Malar J. 2022 Aug 20;21:240. doi: 10.1186/s12936-022-04252-0 (PMC9392271; doi:10.1186/s12936-022-04252-0)
Supplement: Supplementary file 3 — Additional file 3: Table S3. Baseline distribution of study sample characteristics and crude bivariate associations of placental malaria against different explanatory variables among Ghanaian mothers. [file 12936_2022_4252_MOESM3_ESM.docx]

## Additional file 3

**Baseline distribution of study sample characteristics and crude bivariate associations of placental malaria against different explanatory variables among Ghanaian mothers aged 14-49.**

| **Variable** | **Category** | **Total (%)**  **(n=1,823)** | **PM**  **Cases (row %)** | **Crude Odds Ratio**  **(95% CI)** | **p-value**  **(Chi2 )** |
| --- | --- | --- | --- | --- | --- |
| **Gravidity**  **(n= 1,823)** | 4  3  2  1  0 | 554 (30.39)  251 (13.77)  325 (17.83)  341 (18.71)  352 (19.31) | 124 (22.38)  54 (21.51)  121 (37.32)  152 (44.57)  232 (65.91) | 1  0.95 (0.66-1.36)  2.06 (1.52-2.79)  2.79 (2.06-3.77)  6.70 (4.82-9.33) | <0.001 |
| **Gravidity binary**  **(n= 1,823)** | Multigravidae  Primigravidae | 1471 (80.69)  352 (19.31) | 451 (30.66)  232 (65.91) | 1  4.37 (3.38-5.66) | <0.001 |
| **Area of residence**  **(n= 1,823)** | Urban  Rural | 382 (20.95)  1441 (79.05) | 144 (37.70)  539 (37.40) | 1  0.99 (0.79-1.24) | 0.916 |
| **Age group**  **(n=1808)^2^** | <18  18-25  25-49 | 204 (11.28)  672 (37.17)  932 (51.55) | 142 ( 69.61)  304 ( 45.24)  231 ( 24.79) | 1  0.36 (0.26-0.51)  0.14 (0.10-0.21) | <0.001 |
| **Maternal Education level**  **(n= 1,823)** | None  Primary school  Middle school  Secondary school or higher | 500 (27.43)  462 (25.34)  771 (42.29)  90 (4.94) | 168 (33.60)  191 (41.34)  294 (38.13)  30 (33.33) | 1  1.39 (1.07- 1.81)  1.22 (0.96- 1.54)  0.99 (0.61-1.60) | 0.074 |
| **Sickle cell status**  **(n= 784)^2^** | Normal  Carriers  Sickling | 576 (73.47)  148 (18.88)  60 (7.65) | 235 (40.80)  54 (36.49)  24 (40.00) | 1  0.83 (0.57-1.21)  0.96 (0.56-1.67) | 0.630 |
| **ITN usage^1^ ^3^**  **(n= 1,782)** | No  Yes | 861 (48.32)  921 (51.68) | 299 (34.73)  369 (40.07) | 1  1.26 (1.03-1.52) | 0.020 |
| **Season of pregnancy**  **(n= 1,823)** | Low transmission  High transmission | 512 (28.09)  1311 (71.91) | 185 (36.13)  498 (37.99) | 1.0  1.08 (0.88- 1.34) | 0.462 |
| **Distance from nearest health facility**  **(n= 1,823)** | Within 5KM  More than 5KM | 1392 (76.36)  431 (23.64) | 513 (36.85)  170 (39.44) | 1.0  1.11 (0.89-1.39) | 0.332 |
| **Wealth index**  **(n= 1,823)** | Least poor  Less poor  Poor  More poor  Most poor | 364 (19.97)  365 (20.02)  364 (19.97)  365 (20.02)  365 (20.02) | 109 (29.95)  118 (32.33)  136 (37.36)  172 (47.12)  148 (40.55) | 1.00  1.12 (0.82- 1.53)  1.40 (1.02-1.90)  2.08 (1.53-2.84)  1.59 (1.17-2.17) | <0.001 |
| **Mothers relationship status**  **(n= 1,823)** | Married  Living together  Widowed/Divorced/Separated  Single | 906 (49.70)  729 (39.99)  37 (2.03)  151 (8.28) | 259 (28.59)  321 (44.03)  16 (43.24)  87 (57.62) | 1.00  1.97 (1.60-2.42)  1.90 (0.98-3.71)  3.40 (2.36-4.88) | <0.001 |
| **Mothers religion**  **(n= 1,823)** | Catholic  Protestant  Pentecostal  Muslim  Traditional African  Other | 368 (20.19)  515 (28.25)  523 (28.69)  288 (15.8)  54 (2.96)  75 (4.11) | 160 (43.48)  188 (36.5)  189 (36.14)  100 (34.72)  19 (35.19)  27 (36.00) | 1.00  0.75 (0.57-0.98)  0.73 (0.56- 0.97)  0.69 (0.50-0.95)  0.71 (0.39- 1.28)  0.73 (0.44-1.23) | 0.193 |
| **Anaemia status**  **(n= 1,823)** | Yes  No  Unknown | 28 (1.54)  1776 (97.42)  19 (1.04) | 10 (35.71)  669 (37.67)  4 (21.05) | 1.0  1.09 (0.50-2.37)  0.48 (0.12-1.9) | 0.324 |
| **Fansidar (IPTp-SP) given at health facility**  **(n= 1,822)** | Yes  No  Don’t know | 1730 (94.95)  91 (4.99)  1 (0.05) | 658 (38.03)  25 (27.47)  0 (0.0) | 1.0  0.62 (0.39-0.99)  - | 0.090 |
| **Doses of Fansidar (IPTp-SP)^1^ received during pregnancy**  **(n= 1,820)** | 0  1  2  3 | 96 (5.37)  234 (12.86)  459 (25.22)  1031 (56.65) | 27 (28.12)  94 (40.17)  178 (38.78)  383 (37.15) | 1.0  1.72 (1.02-2.89)  1.62 (1.00-2.63)  1.51 (0.95-2.40) | 0.196 |
| **Number of Tetanus toxoid vaccine received during pregnancy**  **(n= 1,822)** | 0  1  2  3  4  5  Don’t know | 380 (20.86)  898 (49.29)  439 (24.09)  61 (3.35)  14 (0.77)  5 (0.27)  25 (1.37) | 113 (29.74)  339 (37.75)  192 (43.74)  22 (36.07)  6 (42.86)  2 (40.0)  9 (36.0) | 1  1.43 (1.11-1.86)  1.84 (1.37-2.46)  1.33 (0.76-2.35)  1.77 (0.60- 5.34)  1.58 (0.26- 9.58)  1.33 (0.57-3.10) | 0.008 |

1 Abbreviations; IPTp, intermittent preventive treatment of malaria in pregnancy; ITN, insecticide-treated bed nets

2 Variables, age group had 15 missing data points and sickle cell group had 1039 missing data points

3 Maternal ITN use was assessed at the last contact with the study mother prior to delivery
