# Supplementary material for: Assessing the relationship between gravidity and placental malaria among pregnant women in a high transmission area in Ghana
Source: Malar J. 2022 Aug 20;21:240. doi: 10.1186/s12936-022-04252-0 (PMC9392271; doi:10.1186/s12936-022-04252-0)
Supplement: Supplementary file 4 — Additional file 4: Table S4. Crude and adjusted odds ratios for the effect of gravidity as a binary variable, age, wealth index and relationship status on placental malaria, adjusted for confounders, estimated by logistic regression. Table S5. Stratum-specific odds ratios from potential effect modifiers identified through Mantel-Haenszel analysis, after assessing for potential confounders. Table S6. Stratum specific odds ratios of effect modifiers of the association between gravidity as a binary variable and placental malaria, estimated by logistic regression fitted with interaction parameters after adjustment for confounders. Gravidity as a binary variable specific results. [file 12936_2022_4252_MOESM4_ESM.docx]

Additional file 4

List of Tables

Table 1. Crude and adjusted odds ratios for the effect of gravidity as a binary variable, age, wealth index and relationship status on placental malaria, adjusted for confounders, estimated by logistic regression

Table 2. Stratum-specific odds ratios from potential effect modifiers identified through Mantel-Haenszel analysis, after assessing for potential confounders

Table 3. Stratum specific odds ratios of effect modifiers of the association between gravidity as a binary variable and placental malaria, estimated by logistic regression fitted with interaction parameters after adjustment for confounders

## Gravidity as a binary variable

The univariate analysis (Additional file 3) showed that gravidity was strongly associated with odds of PM (p<0.001), and compared to multigravidae, primigravidae had higher odds of developing PM. When grouped as a binary variable (primigravidae vs multigravidae), the crude odds ratio for the association of primigravidity and PM was OR=4.37 (95% CI 3.38-5.66, P<0.001).

The final fitted logistic regression model (Table 1) included PM, gravidity, and the confounding variables age, wealth index and relationship status. After adjusting for all confounding variables through forward modelling, there remained strong evidence that primigravidae were at increased odds of developing PM, adjusted OR=2.58 (95% CI 1.90-3.47), N=1808, P<0.001.

**Table 1. Crude and adjusted odds ratios for the effect of gravidity as a binary variable, age, wealth index and relationship status on placental malaria, adjusted for confounders, estimated by logistic regression in Ghanaian women aged 14-49 (N=1,808)**

|  |  | **Crude model** | | **Model adjusted for age ^1^** | | **Fully adjusted model ^2^** | |
| --- | --- | --- | --- | --- | --- | --- | --- |
| **Variable** | **Category** | **OR (95% CI)** | **p-value**  **(LRT)** | **OR (95% CI)** | **p-value**  **(LRT)** | **OR (95% CI)** | **p-value**  **(LRT)** |
| **Gravidity** | MG | *(REF)* |  | *(REF)* |  | *(REF)* |  |
|  | PG | 4.37 (3.38-5.66) | <0.001 | 2.45 (1.83 -3.29) | <0.001 | 2.58 (1.90 -3.47) | <0.001 |
| **Age** | <18 | *(REF)* |  | (REF) |  | *(REF)* |  |
|  | 18-25 | 0.36 (0.26-0.51) |  | 0.55 (0.38-0.80) |  | 0.62 (0.42-0.90) |  |
|  | 25-49 | 0.14 (0.10-0.21) |  | 0.27 (0.18-0.40) |  | 0.33 (0.22-0.50) |  |
| **Wealth index** | Least poor | *(REF)* |  | *-* |  | *(REF)* |  |
|  | Less poor | 1.12 (0.82- 1.53) |  | - |  | 1.29 (0.75-1.47) |  |
|  | Poor | 1.40 (1.02-1.90) |  | - |  | 1.35 (0.97- 1.88) |  |
|  | More poor | 2.08 (1.53-2.84) |  | - |  | 2.06 (1.48-2.86) |  |
|  | Most poor | 1.59 (1.17-2.17) |  | - |  | 1.79 (1.29-2.49) |  |
| **Relationship**  **Status** | Married | *(REF)* |  | *-* |  | *(REF)* |  |
|  | Living together | 1.97 (1.60-2.42) |  | - |  | 1.29 (1.02-1.63) |  |
|  | Widowed/  Divorced/  Separated | 1.90 (0.98-3.71) |  | - |  | 0.96 (0.47-1.98) |  |
|  | Single | 3.40 (2.36-4.88) |  | - |  | 1.43 (0.95-2.17) |  |

LRT; Likelihood ratio test

Abbreviations: MG, multigravidae; PG, primigravidae

^1^ Wealth index and relationship status were not included in model which only adjusted for age

^2^ Model adjusted for age group, wealth index and relationship status.

### Effect modification

The stratum specific odds ratios for gravidity differed by >10% per strata for the variables; ITN use, area, wealth index, and doses of IPTp-SP, with corresponding low p-values from the tests of homogeneity (Table 2). This provided some evidence that the effect of gravidity on PM differed between the different levels of these variables.

**Table 2. Stratum-specific odds ratios from potential effect modifiers identified through the Mantel-Haenszel analysis, after assessing for potential confounders to the association of gravidity and placental malaria among Ghanaian mothers aged 14-49**

| **Variable** | **Category** | **Stratum specific OR (95% CI)** | **p-value (chi2)** | **Chi2 test for homogeneity (p-value)^1^** |
| --- | --- | --- | --- | --- |
| **Wealth index** | Least poor  Less poor  Poor  More poor  Most poor | 2.70 (1.57-4.64)  3.59 (2.09-6.17)  4.86 (2.71-8.73)  8.86 (4.24-18.53)  6.54 (3.31-12.93) | <0.001 | 0.06 |
| **ITN usage** | Don’t use  Use | 5.77 (3.83-8.70)  3.27 (2.33-4.58) | <0.001 | 0.04 |
| **Doses of IPTP3** | 0  1  2  3 | 8.13 (2.03-32.59)  8.69 (3.85-19.58)  5.50 (3.22-9.42)  3.29 (2.37-4.58) | <0.001 | 0.07 |
| **Area** | Urban  Rural | 2.97 (1.80-4.90)  5.01 (3.70-6.79) | <0.001 | 0.08 |

^1^ Homogeneity tests test the null hypothesis that the association between gravidity and PM is the same within each level of the potential effect modifier

The adjusted model was then fitted to allow for an interaction between binary gravidity and each of the four effect modifying variables (Table 3). The association between primigravidity and odds of PM was strongest in rural areas, (OR= 2.87, 95% CI 2.04-4.02), however, the p-value from the LRT (P=0.13) indicated very weak evidence against the null hypothesis of no interaction. When the model was fitted for an interaction between gravidity and wealth index, the association between primigravidity and increased odds of PM was strongest in the two poorest groups of women (OR=5.00, 95% CI 2.47- 10.14) ( OR=3.42 95% CI 1.73- 6.73), LRT P=0.07. The association between primigravidity and PM was also stronger amongst primigravidae who did not use an ITN ( adjusted OR=3.27, 95% CI 2.14-5.01) than primigravidae who did (adjusted OR=1.92, 95% CI 1.31- 2.81), LRT P=0.05. When the model was fitted for an interaction between gravidity and doses of IPTp-SP, the increased odds of PM in PG, relative to MG, was greatest in those who took no IPTp-SP (OR 4.95, 95% CI 1.32-18.53), and smallest in those who took at least 3 IPTp-SP (OR 1.86, 95% CI 1.29-2.70) LRT P= 0.04.

**Table 3. Stratum specific odds ratios of effect modifiers of the association between gravidity as a binary variable and placental malaria among Ghanaian mothers aged 14-49, estimated by logistic regression fitted with interaction parameters after adjustment^1 2^ for confounders**

| **Variable** | **Category** | **Gravidity group** | **% PM (pm/N)** | **Stratum specific OR (95% CI)** | **p-value (LRT)** |
| --- | --- | --- | --- | --- | --- |
| **Wealth index (N=1823)** | Least poor | Multigravidae  Primigravidae | 25.4% (74/291)  47.9% (35/73) | 1  1.59 (0.91-2.78)^2^ | 0.07 |
|  | Less poor | Multigravidae  Primigravidae | 255.8% (43/77)  6.0% (75/288) | 1  2.01 (1.16- 3.50)^2^ |  |
|  | Poor | Multigravidae  Primigravidae | 30.0% (88/293)  67.6% (48/71) | 1  2.83 (1.56- 5.14)^2^ |  |
|  | More poor | Multigravidae  Primigravidae | 38.0% (112/294)  84.5% (60/71) | 1  5.00 (2.47- 10.14)^2^ |  |
|  | Most Poor | Multigravidae  Primigravidae | 33.4% (102/305)  76.0% (46/60) | 1  3.42 (1.73- 6.73)^2^ |  |
| **ITN use**  **(N=1782)** | Don’t use | Multigravidae  Primigravidae | 27.8% (199/716)  69.0% (100/145) | 1  3.27 (2.14-5.01)^1^ | 0.05 |
|  | Use | Multigravidae  Primigravidae | 33.9% (245/723)  62.0% (124/198) | 1  1.92 (1.31-2.81)^1^ |  |
| **Doses of Fansidar (IPTp-SP) (N=1820)** | 0 | Multigravidae  Primigravidae | 21.7% (18/83)  69.2% (9/13) | 1  4.95 (1.32-18.53)^1^ | 0.04 |
|  | 1 | Multigravidae  Primigravidae | 29.5% (54/183)  78.4% (40/51) | 1  4.61 (2.14-9.95)^1^ |  |
|  | 2 | Multigravidae  Primigravidae | 30.9% (114/369)  71.1% (64/90) | 1  3.55 (2.05-6.15)^1^ |  |
|  | 3 | Multigravidae  Primigravidae | 31.7% (264/834)  60.4% (119/197) | 1  1.86 (1.29-2.70)^1^ |  |
| **Area (N=1823)** | Urban | Multigravidae  Primigravidae | 31.5% (92/292)  57% (52/90) | 1  1.82 (1.08-3.09)^1^ | 0.13 |
|  | Rural | Multigravidae  Primigravidae | 30.4%(359/1179)  68.7% (180/262) | 1  2.87 (2.04-4.02)^1^ |  |

LRT; Likelihood ratio test

^1^ Adjusted for age, wealth and relationship status

^2^ Adjusted for age relationship
